# Supplementary material for: Postnatal quality of care measures for mothers and newborns at home: A scoping review
Source: PLOS Glob Public Health. 2024 Aug 20;4(8):e0003498. doi: 10.1371/journal.pgph.0003498 (PMC11335102; doi:10.1371/journal.pgph.0003498)
Supplement: S1 Table — (PDF) [file pgph.0003498.s002.pdf]

Data source	Search string	Hits	Date
PubMed	("postnatal care"[MeSH Terms] OR "postpartum period"[MeSH Terms] OR "postnatal care*"[tiab] OR "postpartum care*"[tiab] OR "postpartum program*"[tiab] OR "postnatal program*"[tiab] OR "postnatal experience*"[tiab]) AND (“home care services”[MeSH Terms] OR “home care*”[tiab] OR “home-based care*”[tiab] OR “home based care*”[tiab] OR “domiciliary care*”[tiab] OR “care at home”[tiab]) AND (2010/01/01:2023/10/31[dp])	144	31/10/’23
Embase	('postnatal care*':ab,ti OR 'postpartum care*':ab,ti OR 'postpartum program*':ab,ti OR 'postnatal program*':ab,ti OR 'postnatal experience*':ab,ti) AND ('home care service*':ab,ti OR 'home care*':ab,ti OR 'home-based care*':ab,ti OR 'home based care*':ab,ti OR 'domiciliary care*':ab,ti OR ‘care at home*':ab,ti)	60	31/10/’23

Web of Science	(TS=("postnatal care" OR "postpartum care" OR "postpartum program" OR “postpartum programme” OR "postnatal program” OR “postnatal programme” OR "postnatal experience" OR “postnatal experiences”))<br><br>AND<br><br>(TS=(“home care service” OR "home care” OR “home-based care” OR “home based care” OR “domiciliary care” OR “care at home”))	57	31/10/’23
