# Supplementary material for: Postnatal quality of care measures for mothers and newborns at home: A scoping review
Source: PLOS Glob Public Health. 2024 Aug 20;4(8):e0003498. doi: 10.1371/journal.pgph.0003498 (PMC11335102; doi:10.1371/journal.pgph.0003498)
Supplement: S2 Table — (PDF) [file pgph.0003498.s003.pdf]

Author	Title	Publication year	Source	Purpose of study	Study design	Geo-graphical location	Content of home-based PNC	Clearly defined measures?	Number of PNC components	Timing and frequency home visits	Quality of care measures	Funding resources	Declared conflicted interests
Aksu et al.	The effect of postnatal breastfeeding education/support offered at home 3 days after delivery on breastfeeding duration and knowledge: a randomized trial	2011	Electronic database	To determine the effects of breastfeeding education/support offered at home on day 3 postpartum on breastfeeding duration and knowledge	Clinical trial	Aydın, Turkey	- Breastfeeding education/support at home based on the WHO/UNICEF breastfeeding counseling/lactation management courses	No	1	1 home visit: on day 3 postpartum	/	Unclear	Not known
Bora Güneş et al.	Effects of a home follow-up program in Turkey for urban mothers of premature babies	2020	Electronic database	To examine the effects of a home follow-up program in Turkey on care problems, anxiety, and depression levels of mothers after the birth of a premature baby	Cross-sectional study	Turkey	- Infant physical evaluation: At each follow-up, infants were physically examined including their body temperature, pulse rate, respiration rate, blood pressure, peripheral saturation, height, body weight, and head circumference<br>- Basic care skills (e.g., changing diapers, diaper rash care, skin care, bathing, dressing, etc.) were performed with mothers to promote appropriate skills<br>- Nursing care: interventions for strengthening sleep, improving participation of the family in care, and supporting caregivers, care for umbilical cord<br>- Emotional and cognitive support: mothers were provided information on baby care, strategies for decreasing anxiety, means for strengthening coping methods, and supportive decision-making	No	7	4 home visits: during week 1, week 2, week 3 and on day 40-42 postpartum	/	Hacettepe Üniversitesi, Grant/Award Number: THD-2016-8472	Not known
Campbell-Voytal et al.	Postpartum doulas: motivations and perceptions of practice	2011	Electronic database	To describe the perceptions of a United States cohort of experienced birth doulas who were among the first [illegible]	Qualitative, longitudinal study	United States	- Supporting women: presence, listening, protection, and translation between parents' expectations and the challenges of integrating a new infant into the family<br>- Taking the mother's perspective: the doula adapted to the mother's perspectives or desires<br>[illegible]	No	3	6 home visits: during the first 12 weeks postpartum	Studies the perceptions of a US cohort of experienced birth doulas who were among the first in the country to be [illegible]	No funding to carry out the research investigation	Not known

				in the country to be trained to provide postpartum support			- Empowering women: sharing information and affirming women's values and opinions so that their transition to motherhood was optimal<br>- Empowering families: flexibility, following family cues, and assisting families to develop parenting skills				trained to provide postpartum support on following areas: program penetration, program quality, family satisfaction and family connections to community resources.		
Dodge et al.	Effect of a community agency-administered nurse home visitation program on program use and maternal and infant health outcomes: a randomized clinical trial	2019	Electronic database	To test the effect of a nurse home visitation program for families with newborns implemented in a community setting on program penetration and fidelity and family outcomes	Randomized clinical trial	North Carolina, United States	Assessment of parent health, infant health, medical home, child care planning, parent-infant relationship, management of infant crying, material supports, family violence, mother's past experience of maltreatment,<br>maternal depression and anxiety, parental substance abuse, and parental social support from others	No	7	1 to 3 home visits	/	Funding was provided by county government, Medicaid reimbursement, and philanthropy	Not known
Fallahnejad et al.	Investigation of the effect of postpartum home visit intervention on promoting mothers' exclusive breastfeeding in Falavarjan, Isfahan Province: clinical trial research	2021	Electronic database	The aim of this study was to investigate the effect of postpartum home visits on exclusive breastfeeding	Clinical trial	Falavarjan, Isfahan Province, Iran	- Creating and strengthening self-esteem and emotional support for mothers<br>- Breast examination in terms of problems and breastfeeding<br>- Breastfeeding training according to the plan<br>- Observing breastfeeding and discovering problems<br>- Emphasizing exclusive breastfeeding<br>- Emphasizing breastfeeding according to the desire and demand of the infant	No	6	4 home visits: on day 3, day 7, day 14 and day 42 postpartum	/	Shiraz University of Medical Sciences	None declared

Forster et al.	The structure and organisation of home-based postnatal care in public hospitals in Victoria, Australia: a cross-sectional survey	2016	Grey literature	To explore the structure and organisation of public hospital home-based postnatal care in Victoria, Australia.	Cross-sectional study	Victoria, Australia	- Maternal observations: maternal vital signs (including pulse, temperature and blood pressure); postnatal observation of lochia, breasts, urinary function, bowels, perineum, and caesarean wound); and measurement of postnatal depression with a psychological/social/behavioural instrument<br>- Neonatal observations: observation of bowel function, urine output, feeding and skin colour; infant weighing; routinely check the umbilical cord<br>- Assessment of the home environment<br>- Assessment of social support<br>- Breastfeeding assessment and support<br>- Referral to community support services<br>- Provision of ongoing education to mother, partner and other family members	Yes	10	Median number of 1 home visit for primiparous women and 2 home visits for multiparous women	/	La Trobe University Faculty Grant	None declared
Gogia et al.	Home-based neonatal care by community health workers for preventing mortality in neonates in low- and middle-income countries: a systematic review	2016	Electronic database	To assess the effect of home-based neonatal care provided by community health workers (CHWs) for preventing neonatal, infant and perinatal mortality in resource-limited settings with poor access to health facility based care	Systematic review	Low- and middle-income countries	- Care of the newborn immediately after birth: keeping the baby warm, neonatal resuscitation (if required) and early initiation of breastfeeding<br>- Health education and/or counseling of families regarding neonatal care practices such as exclusive breastfeeding, keeping the baby warm and hygienic cord care<br>- Education to improve caregiver recognition of life-threatening neonatal problems<br>- Education to improve health care-seeking behaviors<br>- Identification of signs of severe neonatal morbidities and referral to a health facility<br>- Home-based management of neonatal morbidities	No	9	/	/	Department of Maternal, Newborn, Child and Adolescent Health and Development, World Health Organization, Geneva, Switzerland.	Non declared

Goodman et al.	Effect of a Universal Postpartum Nurse Home Visiting Program on Child Maltreatment and Emergency Medical Care at 5 Years of Age: A Randomized Clinical Trial	2021	Electronic database	To determine the effect of randomization to Family Connects on child maltreatment investigations and emergency medical care through 5 years of age	Clinical trial	North Carolina, United States	- Identify family-specific needs, deliver education and intervention, and connect families with community resources matched to their needs<br>- Health care: maternal health, infant health, and health care plans<br>- Parenting and childcare: childcare plans, parent-child relationship, and management of infant crying<br>- Family material resources and safety: material supports, family and community safety, and birthing parent history of parenting difficulties<br>- Parent well-being: mental health, substance abuse, and social-emotional support	No	7	1 to 3 home visits: beginning from 3 weeks postpartum	/	Unclear	Not known
Grover et al.	Evaluation of home based postnatal care provided by accredited social health activists worker in rural community of Haryana: a cross-sectional study	2019	Grey literature	To assess the quality of home-based postnatal care provided by accredited social health activist (ASHA) workers and various factors associated with it.	Cross-sectional study	Haryana, India	- Counselling of the couples to choose an appropriate family planning method, counselling on contraception<br>- Counselling on early identification of postpartum complications (including heavy bleeding, severe pain in abdomen, fever, convulsions or fits, foul smelling discharge, breast nipple problems) and danger signs in new-born and refer appropriately<br>- Counselling on care of cord, eye of newborn<br>- Counselling on diet, hygiene, rest and resumption of sexual intercourse	No	6	- 6 home visits in case of an insititutional birth: on day 3, day 7, day 14 , day 21 and day 28 postpartum<br>- 1 extra home visit in case of a home birth: on day 1 postpartum	The study assesses the quality of HBPNC provided by ASHA workers and various factors associated with it.	No funding sources	None declared
Goyal et al.	Home visiting for first-time mothers and subsequent pregnancy spacing	2017	Electronic database	Determine association of home visiting with subsequent pregnancy outcomes	Retrospective study	Ohio, United States	- Provide pregnancy education and care coordination<br>- Promote a nurturing home environment<br>- Optimize child development<br>- Link families to health services<br>- Promote economic self-sufficiency	No	5	/	/	Supported by the Building Interdisciplinary Research Careers in Women's Health program (5K12HD051953-07), co-funded by the Office of Research on	Non declared

												Women's Health (ORWH) and the Eunice Kennedy Shriver National Institute of Child Health and Human Development (NICHD)	
Harrison et al.	Delivery of home-based postpartum contraception in rural Guatemalan women: a cluster-randomized trial	2019	Electronic database	To observe whether home delivery of the contraceptive implant increases utilization of the device above the baseline rate	Cluster randomized trial	Southwest Trifinio, Guatemala	Home-based contraceptive delivery	No	1	2 home visits: on an unspecified day and on day 40 postpartum	/	Eunice Kennedy Shriver National Institute of Child Health and Human Development Women's Reproductive Health Research Fellowship + University of Colorado School of Medicine, Department of Obstetrics and Gynecology	Non declared

Hodgins et al.	Postnatal care, with a focus on home visitation: a design decision-aid for policymakers and program managers	2017 (latest date of references)	Grey literature	To provide practical operational guidance to ministries of health, policymakers, program managers and other decision-makers on how to design and optimal mix of service delivery approaches forthe postnatal period that is feasible and repsonsive to the particular circumstances and conditions of a given setting	Guideline document	Global	- Group-based health education<br>- Counseling provided during home visits, during pregnancy and the postnatal period<br>- Facilitated referral for newborn illness<br>- Case management of newborn illness at home or the health post	No	3	/	/	United States Agency for International Development (USAID)	Not known
Johansson et al.	Mothers' experiences in relation to new Swedish postnatal home-based model of midwifery care - a cross-sectional study	2019	Electronic database	To describe mothers´ experiences in relation to a new postnatal home-based model of midwifery care	Cross-sectional study	Stockholm, Sweden	- Emotional and informational family support (both parents)<br>- Examinations of the infants' weight, bilirubinemia, hypoglycemia, metabolic screening blood sample test<br>- The new mother received examinations for lacerations and blood pressure, if appropriate	Yes	3	1 to 3 home visits: during first week postpartum	/	No funding to carry out the research investigation	Non declared

Mafubelu	4 Doable actions for mother and newborn care	2012	Grey literature	4 Doable Actions for Mother and Newborn Care	Journal article (UN Chronicle)	Global	Referred to the WHO/UNICEF recommendations:<br>- Keeping the baby warm<br>- Increasing hand washing<br>- Providing hygienic umbilical cord and skin care<br>- Identifying conditions/danger signs requiring additional care and counselling on when to take a newborn to a health facility<br>- Counseling on birth spacing and nutrition<br>- Observing blood flow, measuring temperature and providing iron-folic acid supplementation and contraceptive methods	Yes	10	/	/	United Nations	Not known
Milani et al.	Postpartum home care and its effects on mothers' health: a clinical trial	2017	Electronic database	A comprehensive postpartum home care program was compiled by performing a comparative study and a clinical trial was carried out to assess the effect of home visists on mothers' health	Comparative study and clinical trial	Iran	Examinations, observations, questions and necessary instructions and training with regard to:<br>- personal hygiene<br>- mental, psychological, and sexual health (Edinburgh Postnatal Depression questionnaire was used to screen for postpartum depression)<br>- oral and dental health risk factors<br>- common complaints in the postpartum period<br>- nutrition in this period and use of supplements<br>- breastfeeding and its related problems and duration<br>- care for the infant<br>- contraception<br>- how to manage postpartum complications<br>- exercise activities	No	8	2 home visits: one between day 3-5 and one between day 13-15 postpartum	/	Nil	None declared

Mirmolaei et al.	Comparison of Effects of Home Visits and Routine Postpartum Care on the Healthy Behaviors of Iranian Low-Risk Mothers	2014	Electronic database	To compare the effect of two midwife visits at home to usual postpartum care on the healthy behaviors of low-risk Iranian mothers.	Clincial trial	Iran	- Greeting and establishing an intimate relationship with the mother<br>- Identifying mother's socioeconomic status (SES) and lifestyle<br>- Assessing vital signs, consciousness, alcoholism, convulsion, breathing problems, abdominal or flank pain, any bleeding, suture complications, defection problems, vertigo, inflammation of the gums, shock, symptoms of psychological disorders, comorbidities and medical history<br>- Consultations on family planning (FP), breastfeeding and medicinal supplements<br>- Examination of extremities, breasts, eyes, abdomen and urinary and reproductive organs<br>- Identifying wife and any social abuse and as a main component providing health education based on her SES and health status	Yes	12	2 home visits: between day 10-15 and day 42-60 postpartum	/	Tehran University of Medical Sciences	None declared

Nygren et al.	What's happening during home visits? Exploring the relationship of home visiting content and dosage to parenting outcomes	2018	Electronic database	Determine what home visitors do during home visitis and how much time is being spend on specific topics	Cross-sectional study	Oregon, United States	- Physical health: prenatal health, nutrition, exercise, substance use, smoking<br>- Emotional health: maternal mental health, stress, coping, well-being<br>- Relationships: communication, relationship with partner, domestic violence<br>- Child physical care: physical care of child, breast feeding/nutrition, home safety<br>- Parent-child relationships: attachment, responsiveness, reciprocity, affection, empathy<br>- Early childhood development: temperament, development (social/physical), appropriate expectations<br>- Guidance: modeling, positive discipline, behavior management, routines<br>- Life course: goal setting, family planning, education, employment<br>- Caregiver support: social/parent support, childcare, father involvement, parenting classes<br>- Information/referrals: emergency/crisis plan, housing, utilities	No	12	Average of 28 home visits in this study<br>Average duration of home visit in this study: 67.46 min	/	Health Resources and Services Administration (HRSA) of the U.S. Department of Health and Human Services (HHS) under Affordable Care Act—Maternal, Infant and Early Childhood Home Visiting Program Expansion Grant	None declared

Peterson et al.	Triadic interactions in MIECHV: relations to home visit quality	2018	Electronic database	To relate specific home visiting strategies, namely triadic interactions, with quality ratings of home visits	Cross-sectional study	Iowa, US	Triadic interactions that involve child, parent and home visitor: observing, modeling, coaching<br>This leads to better parent-child relationship and interactions, which is a primary mechanism for improving child development outcomes	No	1	1 home visit<br>Average duration of home visit in this study: 46 min	Studies the rate of quality of home visit practices and engagement based on these areas: home visitor responsiveness to family, home visitor relationship with family, home visitor facilitation of parent–child interaction, home visitor non-intrusive collaboration, parent–child interaction, parent engagement, and child engagement	Us health Resources and Services Administration in collaboration with the Iowa Department of Public Health	Not known
Rahman et al.	Noninstitutional births and newborn care practices among adolescent mothers in Bangladesh	2011	Grey literature	To describe home-based newborn care practices among adolescent mothers and to identify sociodemographic, antenatal care and delivery care factors associated with these practices	Cross sectional study	Bangladesh, India	- Essential newborn care practices: complete cord care, complete thermal protection, and early breastfeeding (within 1 hour after birth)<br>- Preventive check-up (= postnatal care within 24 hours of delivery): whether the respondent used any modern instrument (blade or scissors) to cut the cord, whether the instrument was boiled before use, and whether nothing was applied to the cord, whether the respondent wiped and wrapped the newborn within <10 minutes after birth and whether the newborn was first bathed 72 or more hours following birth	Yes	4	/	/	Unclear	None declared

Sinha et al.	Newborn care practices and home-based postnatal newborn care programme - Mewat, Haryana, India 2013	2014	Electronic database	To study newborn care practices among mothers and describe the knowledge and skills of ASHAs (Accredited Social Health Activists) during home visits	Cross-sectional study	Mewat, India	- Eye care<br>- Kangaroo care<br>- Handwashing with soap and water<br>- Cord care<br>- Exclusive breastfeeding<br>- Delayed bathing<br>- Feeding colostrum<br>- Wrapping the baby in multilayers of cloth (mothers provide it)	No	7	6 home visits: on day 1, 3, 7, 14, 28 and 42 postpartum	Studies the quality of ASHAs' home visits: carrying weighing scale, carrying thermometer, took weight and temperature correctly…	National Rural Health Mission, India	None declared
Stetler et al.	Lessons learned: implementation of pilot universal postpartum nurse home visiting program, Massachusetss 2013-2016	2017	Electronic database	The paper identifies lessons learned from the first 3 years of implementation of "Welcome Family" related to outreach and enrollment, program operations and quality, and follow-up and linkages with community resources	Evaluation study	Massachussets, United States	- Addressing unmet health needs<br>- Addressing breastfeeding<br>- Postpartum depression and social connectedness (screening)<br>- Substance use (screening)<br>- Intimate partner violence (health worker address it at home)<br>- Physical assessment of mother and infant	No	7	1 home visit: in week 2- 4 postpartum	Studies the quality of the universal home visiting program "Welcome Family"	Maternal and Child Health Bureau	None declared

Todd et. al.	Maternal nutrition intervention and maternal complications in 4 districts of Bangladesh: A nested cross-sectional study	2019	Electronic database	To compare the prevalence of antepartum, intrapartum and postpartum complications in mothers who had a nutritional intervention and mothers who didn't	Cross-sectional study	Banglades h	Shasthya Kormi (= trained salaried health workers):<br>- sell micronutrient powders for children<br>- provide supplements for lactating women<br>- provide facility referrals for testing and suspected complications throughout postpartum period<br>Shasthya Shebika (= volunteer community health workers):<br>- home-based counseling<br>- ensuring nutrient intake in meals and intake of micronutrient supplements<br>- identification of any pregnancy-related problems and referral for facility-based care<br>- referrals for care by facility-based or community providers	No	4	4 home visits: within 48 hours, on day 7, 28 and 42 postpartum	/	Alive and Thrive initiative funded by the Bill and Melinda Gates Foundation (Grant No. 50838) + the Global Affairs Canada (Grant No. 7060408)	None declared
Turan et. al.	Development and piloting of a home-based couples intervention during pregnancy and postpartum in southwestern Kenya	2018	Electronic database	To develop and pilot a home-based couples intervention during the postpartum period	Validation study	Southwest Kenya	- infant feeding<br>- family planning<br>- men's health<br>- couple relationship/communication exercise<br>- couple HIV testing and counseling in a health facility (if they wanted and after being informed at home)	No	3	1 home visit: ~1 month postpartum	/	US National Institute of Mental Health (NIMH), through grant R34MH102103	None declared
World Health Organization	WHO technical consultation on postpartum and postnatal care	2010	Grey literature	A revision and update of the WHO guidance for postpartum and postnatal care delivered by skilled providers	Guideline document	Global	- Promote parent- or mother-to-child emotional attachment<br>- Assess relevant safety issues for all family members in the home and environment<br>- Promote safety education and use of basic safety equipment<br>- Maintain a home-based maternal and child health record	No	4	/	Determines the degree of quality evidence.	Unclear	None declared

World Health Organization	Informal meeting on provision of home-based care to mother and child in the first week after birth: follow-up to the Joint WHO	2012	Grey literature	Recommendations for home-based care	Guideline document	Global	- Support to initiate early and exclusive breastfeeding<br>- Support for hygienic cord care<br>- Thermal care: support for keeping the newborn warm<br>- Timely recognition of danger signs<br>- Treatment and referral (to health facility services) when needed	Yes	7	/	Evaluates the quality of PNC home visits and services. It discusses how to improve quality of care.	Unclear	None declared
World Health Organization	WHO recommendations on postnatal care of the mother and newborn	2014	Grey literature	Recommendations for postnatal care of mothers and newborns	Guideline document	Global	- Assessing danger signs and referral to a health facility when needed<br>- Provide care, counselling, advice and medical treatment based on mother's perception of her own health and that of her baby, and based on the health problems she had observed and her actions she had taken in case of symptoms and based on her breastfeeding pattern and what kind of social support she had at home	No	4	- At least 3 home visits: on day 3 (48-72 hours), between day 7-14, and 6 weeks postpartum<br>- 1 extra home visit in case of a home birth: within 24 hours postpartum	In drafting the recommendations, the WHO Steering Group used the evidence summaries for determining the quality of evidence.	Unclear	Not known
World Health Organization	Postnatal care for mothers and newborn - highlights from the 2013 guidelines	2015 a	Grey literature	Highlights of the recommendations for postnatal care of mothers and newborns	Guideline document	Global	- Check-up of clinical signs of severe illnesses: stopped feeding well, history of convulsions, fast breathing, severe chest in-drawing, no spontaneous movement, fever, low body temperature, any jaundice in first 24 hours of life, yellow palms and soles at any age<br>- Continue to promote early and exlusive breastfeeding<br>- Cord care<br>- Reinforce delayed bathing, skin-to-skin contact and immunization	Yes	8	- At least 4 home visits: within 24 hours, on day 3, between day 7- 14, and 6 weeks postpartum<br>- In case of a home birth: first home visit as early as possible within 24 hours, and extra home visit at 24- 48 hours	Takes into account quality of care, for example, ensuring respectful, women-centred quality care is provided for all births.	United States Agency for International Development (USAID)	Not known

World Health Organization	Caring for the newborn at home - participant's manual	2015 b	Grey literature	To educate community health workers on the specific measures that they need to perform during home-based postnatal care visits and to train them how to do it correctly	Guideline document (participant's manual: training course for community health workers)	Multicountry (India and Ghana)	- Hand washing skills<br>- Support of breastfeeding: support the initiation, attachment and positioning<br>- Assess for danger signs: not able to feed or stopped feeding well, convulsions, fast breathing, chest indrawing, high or very low temperature, yellow soles, movement, local infection, measure birth weight and identify small babies	Yes	5	/	/	Unclear	Not known
World Health Organization	Planning handbook for programme managers and planners - caring for newborns and children in the community	2015 c	Grey literature	To inform managers and planners about caring for newborns and children in hte community, to guide thein selecting the best interventions and packages and to guide them in through key decisions and actions in planning and implementing the packages in the context of current country activities	Guideline document (planning handbook)	Multicountry (Zambia and Liberia)	- Assess a newborn for danger signs<br>- Measure weight to identify small baby<br>- Support the mother to initiate and sustain exclusive breastfeeding: assess attachment and suckling and help her to improve position and attachment if necessary<br>- Advise families on optimal care practices fro the newborn: exclusive breastfeeding, keeping the newborn warm, using good hygiene to prevent infections and watching for signs of illness<br>- Identify when a newborn or woman needs referral and assist the family in going to a health facility<br>- Assist families to provide extra care for the small baby including frequent feeding and skin-to-skin care	No	11	3 home visits: on day 1, 3 and 7 postpartum<br><br>For small babies: 2 extra visits on day 2 and 14	Studies strength of implementation of interventions including quality. Studies quality of performance of CHWs, quality of training…	Unclear	Not known

World Health Organization	WHO recommendations on maternal and newborn care for a positive postnatal experience	2022	Grey literature	To provide a comprehensive set of recommendations for care during the postnatal period, focusing on the essential package that all women and newborns should receive with attention to the quality of care, that is provision and experience of care	Guideline document	Global	- Involvement of men in postnatal care and maternal and newborn health<br>- Use of home-based records<br>- Assessment of the home environment<br>- Assessment of the woman and newborn's physical well- being<br>- Assessment of the woman's emotional well-being<br>- Health education<br>- Counselling<br>- Breastfeeding promotion and support	No	9	At least 3 home visits: between 48 and 72 hours, between 7 and 14 days, during 6th week postpartum<br><br>1 extra home visit in case of a home birth: as early as possible within 24 hours postpartum	Performs quality appraisal of the evidence.	USAID and the UNDP-UNFPA-UNICEF-WHO-World Bank Special Programme of Research, Developmentand Research Training in Human Reproduction (HRP)	None declared
Yonemoto et al.	Schedules for home visits in the early postpartum period	2021	Electronic database	To assess the effects of different home-visiting schedules on maternal and newborn mortality during the early postpartum period	Systematic review	high- and low-resource settings	- Breastfeeding and hygiene education<br>- Parenting and child health instruction<br>- General support to families<br>- Attention to early initiation of breastfeeding, exclusive breastfeeding, skin-to-skin contact, delayed bathing, attention to hygiene (e.g. hand washing and water quality), umbilical cord care, infant skin care<br>- Assessment of the mother and newborn, health education, infant feeding support, emotional or practical support and, if necessary, referral to other health professionals or agencies<br>- Assessment of maternal mental health, family circumstances and the home environment	No	16	/	/	National Institute for Health Research (NIHR), via Cochrane Infrastructure funding to Cochrane Pregnancy and Childbirth	None declared
