# Supplementary material for: Postnatal quality of care measures for mothers and newborns at home: A scoping review
Source: PLOS Glob Public Health. 2024 Aug 20;4(8):e0003498. doi: 10.1371/journal.pgph.0003498 (PMC11335102; doi:10.1371/journal.pgph.0003498)
Supplement: Attachment — Submitted filename: Review - comments to authors.pdf [file pgph.0003498.s004.pdf]

Review

I appreciate the opportunity to review the manuscript entitled : “*Postnatal quality of care measures for mothers and newborns at home: a scoping review*”. This scoping review scans the published and grey literature globally to document elements of content of postnatal care provided in the home environment, using the WHO PNC recommendations as a reference framework. The manuscript is well and concisely written and it focuses on an understudied topic, making it a valuable addition to the literature. However, some comments and clarifications remain to be provided; detailed below as major and minor recommendations:

Major comments:

- The results display and describe lists of components/elements of home-based PNC rather than exploring elements of quality of care. Quality of care goes beyond the elements of care/packages of care to include issues such as respectful and person-centred care, trained and skilled care providers, resource availability, etc. These aspects are not captured in the current manuscript and I therefore invite the authors to justify/reflect on the use of the term “quality of care” throughout the manuscript and in the definition of the objective and research question.

- There is a missed opportunity to extract data on the type/cadre of care providers involved in home-based PNC provision and the qualifications ; and the frequency, duration and timing of the home visits which are integral factors to defining PNC provision models.

- The extracted results could also be further aggregated by considering whether these elements of care were provided after home-birth or following discharge from a health facility after a facility-based birth. This nuance is important to allow a better understanding of the rationale for the offer of care elements (e.g. early initiation of BF is likely to be recommended either during ANC or immediately after birth; in the context of home-based PNC, this would only be applicable if birth occurred at home). Additionally, this statement: “*In the postnatal period support is crucial, from both health professionals and family members (49–51). Our review shows home-based PNC is oriented towards providing this social and emotional support and that quality of care measures for home-based PNC focus less on the biomedical aspects of care*.” Although I agree that this is true because of the inherent social support needs during the postpartum period, however these needs are arguably more dominant in the extended postnatal period, whereas the immediate postpartum period requires a higher level of clinical and biomedical aspects of care to the mother and the baby – and whether immediate PNC is provided at home depends on the place of birth.

- There is a discrepancy in the definitions of a core concept:

    - In the introduction line 53: “*PNC at home includes professional care, self-care and family care, contributing to maternal and newborn health and well-being*.”
    - Table 1: “*Home based postnatal care, which is defined as all health care provided to mothers, their partners, newborns, parents/caregivers and families during the six weeks after childbirth at home*.”

  The second definition in Table 1 limits the concept to healthcare ignoring other aspects of care that could be need/provided during the postpartum period e.g. support in domestic chores, social care, childcare for other kids in the family, etc. which are more covered in the first definition and evident in the results as well.

- I was surprised about not seeing any publications from the European context, e.g. the Netherlands, Austria, etc. where postnatal home visits are common practice. (from a quick search on google scholar I founds this paper : https://bmjopen.bmj.com/content/11/9/e046696 where authors define elements of PNC home visits in box 1). Another missing reference from Gaza: https://iris.who.int/handle/10665/352829

- Suggestion instead of figure 2 is to design a comprehensive visual of the elements in the WHO guidelines, highlighting the ones identified in the literature, including the “additional” or “unique” elements that are not in the WHO recommendations. This would help to guide the reader through the landscape and scope of content of PNC in the published literature vis-à-vis the global recommendations and guidelines.

Minor comments:

Methods:
- Ensure use of past tense when narrating the methods. (e.g. in line 81-84).

Results:
- It would nice to mention and describe the total number of elements/components of PNC in each study.

- Table 2 refers to a result on "data sources" however this was not defined in the methods and at this point it is not clear what this indicator aims to describe? The source of data for the paper or for defining the quality measures? and please elaborate how the data source could be facility-based records for getting data on home-visits?

- Table 4- the row on breastfeeding seems to be kind of misplaced?

- Line 182-183: authors used the term 'men's involvement' whereas the content is more relevant to family involvement (beyond only the male parent). It is a bit confusing how this is different from what's been included in the social/emotional support domain of the unique measures in Table 6. Overlap of these two: in Table 6 *"Involvement of men in PNC and maternal and newborn health (4)"*

- Table 6 includes the concept of referral of newborn as a unique measure that is not listed in the WHO guidelines, however, it is not the case: WHO recommendation n25 lists: "The following signs should be assessed during each postnatal care contact, and **the newborn should be referred for further evaluation if any of the signs is present**: not feeding well; history of convulsions; fast breathing (breathing rate > 60 per minute); severe chest in-drawing; no spontaneous movement; fever (temperature > 37.5 °C); low body temperature (temperature < 35.5 °C); any jaundice in first 24 hours after birth, or yellow palms and soles at any age." The referral of the mother is lacking from the WHO and from the literature if I am seeing correctly…

- The discussion lacks a deeper interpretation of the fact that some crucial elements that are missing from the WHO recommendations; e.g. skin-to-skin, identification of abuse against women, referral of women in case of complications, etc.
