# Supplementary material for: Postnatal quality of care measures for mothers and newborns at home: A scoping review
Source: PLOS Glob Public Health. 2024 Aug 20;4(8):e0003498. doi: 10.1371/journal.pgph.0003498 (PMC11335102; doi:10.1371/journal.pgph.0003498)
Supplement: Attachment — Submitted filename: Response to reviewers home-based PNC.docx [file pgph.0003498.s005.docx]

Dear Reviewers and Editor,

Thank you for thoroughly reviewing the manuscript and providing constructive feedback. We have carefully reviewed and addressed each of your comments, making the necessary corrections to the manuscript. All changes have been saved under track changes, and this document is provided along with this letter. The page numbers mentioned below refer to the final pdf-document.

We would also like to request the Editor to grant first co-authorship to Ann-Sofie Mespreuve and Lise Apers for this scoping review, as we were unable to indicate this during the PLOS Global Public Health submission process. Currently, only one first author is registered.

**Reviewer 1**

1. **The definition of the content of PNC starting in the introduction is very brief and should be expanded. Please reference the new guidelines here and specify some of the differences in facility and home-based PNC. This should be used to explain why different measures for facility and home PNC are needed i.e. is the content and approach different or is the difference more the location of the care?**

A sentence was added in the introduction (line 57, page 3) showing home-based care has unique components, including assessment of the home environment, social and emotional support, and empowerment of mothers and their families: “*Some components characterizing PNC in the home-based context include assessment of the home environment, social and emotional support, and empowerment of mothers and their families.”*

2. **Clarify if the review focuses on measures of recommended practices or on any measure of postnatal care? For example, does the review include measures used in studies testing new content or approaches?**

This review focuses on any measure of postnatal care, not only the recommended measures and/or practices. A sentence has been added to the eligibility criteria to make this clearer (line 90, page 5): “*Moreover, articles have been included that cover any care component of home-based PNC, which has a broader scope than just recommended measures or practices. While validity and utility studies were considered, they were not the main focus of this scoping review.”*

3. **In the introduction the authors talk about the growing importance of home visits for PNC, please back this up with coverage data and describe other strategies for PNC such as outpatient visits.**

Some additional articles were referenced in the introduction about (cost-) effectiveness data of home-based PNC and outpatient visits to describe other strategies for PNC (line 52, page 3): “*Therefore, utilization of outpatient health services or home visits by trained health professionals are becoming increasingly important to ensure quality PNC (10). PNC at home includes professional care, self-care and family care, contributing to maternal and newborn health and well-being (7). Home-based PNC has been shown to significantly reduce neonatal mortality, increase rates of exclusive breastfeeding, and provide a cost-effective way to improve newborn health outcomes*. *(11).”*

4. **I would like to see a clearer research question that helps the reader understand the scope and focus of the review a little more. Is the aim to map out the core domains being measured or to say something about validity and utility?**

The aim of this scoping review is to explore the care components and associated quality measures, rather than assessing the validity and utility of these measures. A sentence at the end of the introduction has been rephrased to clarify this (line 61, page 4): “*This scoping review aims to provide an overview of the existing home-based PNC measures documented in peer-reviewed articles and grey literature in order to explore the care components and the associated quality measures.”*

5. **My understanding is that the authors have mapped any measure that could have been proposed in international guidelines, used in a trial, used in a cross-section study measuring quality of existing services or that were the explicit focus of validation studies?**

As mentioned in the previous comment, we have indeed mapped any home-based postnatal care measure that was found in scientific and grey literature but we have not explicitly focused on validation studies. A few articles were included that conduct a validity assessment but this has not been the main focus of the scoping review. We have clarified the objective of this study in the introduction and have added a sentence in the eligibility criteria to further elaborate on this (line 61, page 4): “*This scoping review aims to provide an overview of the existing home-based PNC measures documented in peer-reviewed articles and grey literature in order to explore the care components and the associated quality measures.”* and (line 90, page 5): “*Moreover, articles have been included that cover any care component of home-based PNC, which has a broader scope than just recommended measures or practices. While validity and utility studies were considered, they were not the main focus of this scoping review.”*

6. **When they say measure are they referring to an indicator or also to items on an instrument that could be used for a composite measure?**

The measures were separately listed. We do not refer to items on an instrument used for composite measures.

7. **It would be good to clarify the definition of quality used in the data extraction process. Specifically, where did they draw the line between coverage and quality measures? For example, would a measure of the % of women who reported counselling be included or would the measure have to be the % women who had a ‘quality” home visit?**

We based our data extraction on the WHO quality of care framework. Within this framework, coverage is part of quality of care. Therefore, the measures included could either of the examples above. However, when reviewing the literature, the reported measures for quality of care were limited. Therefore, our scoping review is more oriented towards coverage. The extent to which quality of care is discussed in each article has been disclosed in the “S3 Table. Data extraction form”.

8. **In the method section please provide more details as to what a reproducible measure refers to. I found the reference cited too broad to understand how decisions on this were made.**

The measure was defined as not clearly defined (and not reproducible) if you would not be able to re-use the measure in other studies. We agree that this is subjective and has therefore been added under the limitations section (line 277, page 25): “*Furthermore, we acknowledge that the classification of measures identified in the included articles as clearly defined or not is inherently subjective.”*

**A second major comment is around the identified measures and how they are described in the Tables.**

9. **The identified measures in the Tables are very vague. For example, what constituted assessment of maternal health? What is a measure to optimize child development. At the moment, these Tables are too cryptic and the measures need to be re-assessed, re-classified and edited for consistency.**

We acknowledge that the measures are very vague. However, we copied the wording of the measures exactly from original articles, which is illustrated in the “S3 Table. Data extraction form”. The vagueness of the measures is now also incorporated as a finding in the discussion section (line 229, page 24): “*Notably, the majority of measures discussed in this review were described vaguely, suggesting that the published information is not comprehensive enough to be reproducible.”*

10. **When the authors say ‘Assessment of XYZ” is this a measure of whether an assessment occurred or not? Or something about the quality of the assessment? I found it hard to understand what was actually being measured from the Tables.**

We acknowledge that quality of care is indeed very broad, it includes both coverage of interventions and whether interventions were performed according to certain standards. In the tables, we refer to whether an assessment occurred or not, as the aim of the scoping review is to give an overview of the care components and quality of care measures. Detailed information about the quality of care components categorized by the specific articles can be found in the “S3 Table. Data extraction form”.

11. **What is the difference between as assessment and an observation.**

The wordings used to describe the measures included in the tables were copy pasted from the original articles. We decided to cite them and therefore, we cannot change their wordings. In our opinion, assessment and observation can be used interchangeable.

12. **In Table 4 under assessment they have body weight and under observation infant weight. I was unclear what the difference was?**

Once again, these words were copy pasted from the original articles. The specific article that mentioned 'body weight' specified that it was part of an infant's physical evaluation. Therefore, one can assume body weight and infant weight are referring to the same measurement.

13. **I was unclear why thermal care, early breastfeeding, parent-child relationships (and a few others) were included as “unique measures” as there are guidelines on these and they were included in other ways in the Tables.**

The themes discussed in the “unique measures” table do not correspond completely to the guidelines of the WHO and are therefore listed separately. For example, the WHO guidelines emphasize exclusive breastfeeding, while the "unique measures" table focuses on early breastfeeding. Exclusive breastfeeding means only breast milk is given, whereas early breastfeeding refers to breastfeeding within the first hours after birth.

Additionally, for the other measures, we personally do not see them fit within the categories of the WHO guidelines.

14. **I was unsure how data from health facility records (Table 2) were eligible – were these supervision record of home visitors or home visitor records collated and stored in the facility?**

Thank you for this helpful comment. We acknowledge that data from health facility records are not eligible for this scoping review and that we have made a mistake in naming this category. Upon checking the data source of this particular article, we found that the ‘Welcome Family database’ was the primary source of data. We had miscategorized this as “health facility records”, but we have now corrected it to “health program records”.

**Some more specific comments are:**

15. **Under the unique measure section the authors specify that measures outside of the recommendations “characterize home based PNC”. Can they clarify what they mean by this? Are many countries adding additional items to home visits or is this because trials were included which had a wide range of content rather than the content of national programmes? Are these data biased by one or two countries?**

It has been specified that the “unique measures” section characterizes home-based PNC since WHO guidelines, while providing general recommendations for postnatal care, tend to focus more on facility-based care.

The articles identifying “unique measures” cover a wide range of content and geographical scope. Some WHO guideline documents focus on multiple countries and regions with a broad scope, while certain national programs or studies concentrate on specific countries. The overall geographical scope of these studies is extensive, including countries such as Iran, the United States, Australia, Turkey, Sweden, India, Kenya and Bangladesh. The geographical focus of each article is detailed in the “S3 Table. Data extraction form”.

16. **I do not feel the conclusion that home PNC is oriented to social and emotional support is supported by the evidence in the results. First, there are 10 studies alone with umbilical cord care measures and 13 on social/emotional support. Plus, the fact that something is measured does not mean that care is more oriented to that area- it just reflects what is being measured/is measurable. Also, this is a scoping review and many studies are missed so I would not draw such strong conclusions.**

This is a justified observation. We note that, compared to facility-based PNC, which primarily focuses on clinical and biomedical aspects of care, home-based PNC also addressed non-clinical aspects. The particular sentence has been written differently to make the conclusion less strong (line 232, page 24): “*Comparing our results with another recent review on postnatal quality of care measures (not focused on a home-based setting) (7), we found that in addition to the clinical and biomedical aspects of PNC, care at home is also oriented towards social and emotional empowerment, assessment of the home setting, and stimulating the parent-child relationship.”*

17. **As far as I can see there are no experience of care measures- is that correct?**

This is correct. Our scoping review did not have enough search terms (e.g. satisfaction with care, person-centeredness, ...) to focus on experience of home-based care. Therefore, further research is needed to assess experiences of care measures. We acknowledge this is a limitation and added a sentence addressing this in the limitation section of our scoping review (line 275, page 25): “*In addition, we decided to focus on the provision aspect of the quality of care framework, therefore, further research is needed to unravel the experience of home-based PNC measures.”*

18. **The authors need to acknowledge the variation in contexts in the discussion.**

The discussion section was updated to acknowledge the variation in frequency, content and measures for home-based PNC across different contexts (line 225, page 24): “*The frequency, content and measures for home-based PNC varied across the different contexts.”*

**Reviewer 2**

**Major comments:**

1. **The results display and describe lists of components/elements of home-based PNC rather than exploring elements of quality of care. Quality of care goes beyond the elements of care/packages of care to include issues such as respectful and person-centred care, trained and skilled care providers, resource availability, etc. These aspects are not captured in the current manuscript and I therefore invite the authors to justify/reflect on the use of the term “quality of care” throughout the manuscript and in the definition of the objective and research question.**

We reviewed all articles to determine if we missed quality of care measures. A few quality of care measures were found but they were rather limited. Most scientific literature primarily examines the impact of home-based PNC measures on the health outcomes of mothers and newborns to demonstrate the benefits of providing PNC at home. An overview of the quality of care components has been added to the “S3 Table. Data extraction form”. Additionally, we have addressed this issue in the discussion (line 230, page 24): *“Moreover, it was noted that quality of care indicators, such as person-centered care and respectful care, were discussed limitedly in the included studies.”*

2. **There is a missed opportunity to extract data on the type/cadre of care providers involved in homebased PNC provision and the qualifications; and the frequency, duration and timing of the home visits which are integral factors to defining PNC provision models.**

We recognize the missed opportunity in this scoping review. However, the type/cadre of care providers are mostly not described in the original articles. Furthermore, it would be very difficult to compare this across countries as training and what they are allowed to do often differs (e.g. prescribing antibiotics). This would be worth another study but is out of scope for this study.

Regarding the timing, frequency, and duration of PNC provision models, we acknowledge that omitting these factors was an oversight on our part. Consequently, we reviewed all included articles to identify these factors. An additional column was added to the "S3 Table. Data extraction form" to provide an overview of this information and a few lines have been added to the discussion (line 225, page 24): “*The frequency, content and measures of home-based PNC differed across the different contexts. Within individual studies, the frequency of home visits varied according to the study design, ranging from one to 28 visits. According to WHO recommendations, three to five PNC contacts are advised for facility-based deliveries, with an additional contact recommended for home births.”*

3. **The extracted results could also be further aggregated by considering whether these elements of care were provided after home-birth or following discharge from a health facility after a facility-based birth. This nuance is important to allow a better understanding of the rationale for the offer of care elements (e.g. early initiation of BF is likely to be recommended either during ANC or immediately after birth; in the context of home-based PNC, this would only be applicable if birth occurred at home). Additionally, this statement: “In the postnatal period support is crucial, from both health professionals and family members (49–51). Our review shows home-based PNC is oriented towards providing this social and emotional support and that quality of care measures for home-based PNC focus less on the biomedical aspects of care.” Although I agree that this is true because of the inherent social support needs during the postpartum period, however these needs are arguably more dominant in the extended postnatal period, whereas the immediate postpartum period requires a higher level of clinical and biomedical aspects of care to the mother and the baby – and whether immediate PNC is provided at home depends on the place of birth.**

Thank you for this suggestion. We have reviewed all articles to distinguish between measures that apply exclusively for post-facility births and measures after births in general, including home births. This has been clarified in the method section (line 129, page 6): *“A distinction was made between measures intended exclusively for post-institutional births and PNC measures following home births. When the articles did not specify, it was assumed that the measures applied to both institutional and home births.”* Furthermore, the measures involving home births were indicated with an asterisk in all tables in the result section.

4. **There is a discrepancy in the definitions of a core concept: o In the introduction line 53: “PNC at home includes professional care, self-care and family care, contributing to maternal and newborn health and well-being.” o Table 1: “Home based postnatal care, which is defined as all health care provided to mothers, their partners, newborns, parents/caregivers and families during the six weeks after childbirth at home.” The second definition in Table 1 limits the concept to healthcare ignoring other aspects of care that could be need/provided during the postpartum period e.g. support in domestic chores, social care, childcare for other kids in the family, etc. which are more covered in the first definition and evident in the results as well.**

Thank you for drawing our attention to the discrepancy in the definitions. The second definition in Table 1 (line 77, page 4) was updated to include other aspects of care besides health care, including support in household, hygiene and psycho-social wellbeing: *“Home based postnatal care, which is defined as all health care provided to mothers, their partners, newborns, parents/caregivers and families during the six weeks after childbirth at home. This involves medical care, but also non-medical assistance, including support in household, hygiene and psycho-social wellbeing.”*.

5. **I was surprised about not seeing any publications from the European context, e.g. the Netherlands, Austria, etc. where postnatal home visits are common practice. (from a quick search on google scholar I founds this paper: https://bmjopen.bmj.com/content/11/9/e046696 where authors define elements of PNC home visits in box 1). Another missing reference from Gaza:** [**https://iris.who.int/handle/10665/352829**](https://iris.who.int/handle/10665/352829)

Thank you for the excellent suggestions. We acknowledge home-based care is common in Europe, but often not a focus of quality of care studies. The first article does not include the keywords in the abstract and has little focus on home-based care, which explains why it did not appear in our search. The second article is indeed very interesting; however, we decided not to include it in our scoping review because the result and discussion sections primarily focus on the experiences of mothers rather than the content of provided home-based PNC. As we argue in comment number 17, the WHO’s quality of care framework consists of two aspects: the provision and experiences of care. For this scoping review, we are focusing primarily on the content of home-based PNC and quality of care measures.

6. **Suggestion instead of figure 2 is to design a comprehensive visual of the elements in the WHO guidelines, highlighting the ones identified in the literature, including the “additional” or “unique” that are not in the WHO recommendations. This would help to guide the reader through the landscape and scope of content of PNC in the published literature vis-à-vis the global recommendations and guidelines.**

We understand the added value of such a figure. Yet, since there are 53 recommendations, the information is too extensive to create a clear and organized overview. Nonetheless, we thank you for the suggestion.

**Minor comments:**

7. **Ensure use of past tense when narrating the methods. (e.g. in line 81-84).**

The method section was reviewed and it was ensured that past tense was used when narrating the methods.

8. **It would be nice to mention and describe the total number of elements/components of PNC in each study.**

The “S3 Table. Data extraction form” gives an overview of the measures defined in each article. An additional column was added, defining the total number of components of PNC in each study.

9. **Table 2 refers to a result on “data sources” however this was not defined in the methods and at this point it is not clear what this indicator aims to describe? The source of data for the paper or for defining the quality measures? and please elaborate how the data source could be facility-based records for getting data on home-visits?**

This indicator describes the source of data of the original studies. We acknowledge this might not be clear when reading the scoping review and have therefore rephrased the sentences that addressed this information in the result section (line 151, page 7): *“Most of the studies relied on data collected through observations (n=15; 48%) and five used a combination of data sources (16%). Only one study used health program records (3%) and another one used population-based household data (3%). For nine studies, the data source of the original study was either not applicable (e.g. guideline documents) or not defined by the authors (29%).*”. Also the wording in Table 2 (line 158, page 8) has been changed to: “*Data source(s) of the original studies*”. In addition to this, as replied to a comment made by reviewer 1, we made a wrong decision when naming the particular category “health facility records” and have changed the category name to “health program records”.

10. **Table 4- the row on breastfeeding seems to be kind of misplaced?**

Table 4 is based on WHO guideline recommendations and in these recommendations, breastfeeding and exclusive breastfeeding are falling under 'Newborn assessment’. As we based the table on the existing WHO framework, the placement of the ‘Breastfeeding’ row has not been our decision.

11. **Line 182-183: authors used the term ‘men’s involvement’ whereas the content is more relevant to family involvement (beyond only the male parent). It is a bit confusing how this is different from what’s been included in the social/emotional support domain of the unique measures in Table 6. Overlap of these two: in Table 6 “Involvement of men in PNC and maternal and newborn health (4)”**

Thank you for drawing our attention to this. This comment is more than valid and we have adapted the categorization accordingly. Two articles that are dealing with men’s involvement specifically have been kept in Table 5 “Men’s Involvement in PNC”. The three other articles have been added to Table 6 “Social/emotional support and empowerment”.

12. **Table 6 includes the concept of referral of newborn as a unique measure that is not listed in the WHO guidelines, however, it is not the case: WHO recommendation n25 lists: “The following signs should be assessed during each postnatal care contact, and the newborn should be referred for further evaluation if any of the signs is present: not feeding well; history of convulsions; fast breathing (breathing rate > 60 per minute); severe chest in-drawing; no spontaneous movement; fever (temperature > 37.5 °C); low body temperature (temperature < 35.5 °C); any jaundice in first 24 hours after birth, or yellow palms and soles at any age.” The referral of the mother is lacking from the WHO and from the literature if I am seeing correctly…**

Thank you for bringing this to our attention as we indeed did not distinguish between referral of the newborn or referral of the mother. We have renamed the category in Table 6 from “*Referral to health facility when necessary*” to “*Referral of mother to health facility when necessary*” and have reviewed all articles to ensure they addressed referral of the mother. Five articles that address solely the referral of the newborn were reclassified under Table 5, “Assessment of the newborn for danger signs”. Seven articles were retained as they addressed referral of the mother or referral of both the mother and newborn. In the latter case, the articles were also added to Table 5 “Assessment of the newborn for danger signs”.

13. **The discussion lacks a deeper interpretation of the fact that some crucial elements that are missing from the WHO recommendations; e.g. skin-to-skin, identification of abuse against women, referral of women in case of complications, etc.**

Discussion has been added which note that we have found unique measures of care that were not mentioned in the 2022 WHO PNC guidelines (line 257, page 25): “*This scoping review identified several unique measures not included in the 2022 WHO PNC Guideline recommendations, such as social/emotional support and empowerment, assessment of home environment and context, and referral of the mothers to the health facility when necessary. These findings suggest that the WHO guidelines may not be sufficiently oriented towards home-based settings*.” We suggest that the WHO are not sufficiently oriented towards PNC in home-based settings. The WHO recommendations encompass certain guidelines applicable to a home-based setting, yet home-based PNC extends beyond these, encompassing assessments such as evaluating the home environment.
