# Supplementary material for: Postnatal quality of care measures for mothers and newborns at home: A scoping review
Source: PLOS Glob Public Health. 2024 Aug 20;4(8):e0003498. doi: 10.1371/journal.pgph.0003498 (PMC11335102; doi:10.1371/journal.pgph.0003498)
Supplement: Attachment — Submitted filename: Second response to reviewers letter.docx [file pgph.0003498.s006.docx]

Dear Reviewers and Editor,

Thank you for reviewing the revised manuscript and providing additional comments. We have addressed each of these comments, making the necessary corrections to the manuscript. All changes have been saved under track changes, and this document is provided along with this letter. The page numbers mentioned below refer to the final manuscript clean version.

We would also like to request the Editor to grant first co-authorship to Ann-Sofie Mespreuve and Lise Apers for this scoping review, as we were unable to indicate this during the PLOS Global Public Health submission process. Currently, only one first author is registered.

**Journal Requirements**

1. **Please amend your online Financial Disclosure statement. If you did not receive any funding for this study, please simply state: “The authors received no specific funding for this work.”**

We amended the wording of the Financial Disclosure statement in the online submission forum to: “The authors received no specific funding for this work.”

2. **Please update your online Competing Interests statement. If you have no competing interests to declare, please state: “The authors have declared that no competing interests exist.”**

We amended the wording of the Competing Interests statement in the online submission forum to: “The authors have declared that no competing interests exist.”

3. **Please upload your main article file as a .doc, .docx or .rtf file.**

The revised manuscript with track changes and the clean version of the manuscript were both uploaded as a .docx file.

4. **Please provide separate figure files in .tif or .eps format only and remove any figures embedded in your manuscript file. Please also ensure that all files are under our size limit of 10MB. You may leave the figure captions or legends in the manuscript.**

Figure 1 and 2 are removed from the manuscript and converted to a .tif file, which can be found in the online submission forum. The figure captions can be found in the manuscript.

**For more information about how to convert your figure files please see our guidelines:** [**https://journals.plos.org/globalpublichealth/s/figures**](https://journals.plos.org/globalpublichealth/s/figures)

5. **Please review your reference list to ensure that it is complete and correct. If you have cited papers that have been retracted, please include the rationale for doing so in the manuscript text, or remove these references and replace them with relevant current references. Any changes to the reference list should be mentioned in the rebuttal letter that accompanies your revised manuscript. If you need to cite a retracted article, indicate the article’s retracted status in the References list and also include a citation and full reference for the retraction notice.**

No changes to the reference list were made.

**Reviewer 2**

1. **The response letter clarifies that the focus of the paper is on the provision aspect rather than the experiences - this point should be mentioned clearly in the objectives; the definition of the concept in Table 1, and in the methods. Having the following sentence in the “information resources” section could otherwise be misleading to readers: “data were included when the topic covered any quality of care measure of PNC in a home-based setting”, particularly if studies included content on experiences of care were systematically excluded from the review (same as was applied to the study from Gaza in the response letter). Having said that, I remain uncertain about whether the lack of including results/papers on experiences of care was a choice of the authors or was a finding of the scoping review literature, as the response letter also includes this: “Moreover, it was noted that quality of care indicators, such as person-centered care and respectful care, were discussed limitedly in the included studies.” Making the lack of information on experiences of care a possible finding of the review. This distinction should be clearly clarified in the objectives and methods.**

The lack of results on experiences of care was a choice of the authors. The focus of this scoping review was specifically on provision of care. Therefore, as suggested by the reviewer, we updated the objective to make this clearer (line 61, page 4): *“This scoping review aims to provide an overview of the existing home-based PNC measures documented in peer-reviewed articles and grey literature in order to explore the care components and the associated quality measures. The focus of this review lays on the provision of care rather than the experience of care.”* Additionally, the methods section on “Information sources” was updated to mention this specifically (line 88, page 5): *“data were included when the topic covered any quality of care measure related to the provision of PNC in a home-based setting. Therefore, sources that focused exclusively on the experience of care or on provision of care solely possible in a facility-based setting were excluded”*

Table 1 on the concept of home-based PNC was not adapted, as this refers to the definition of home-based PNC.

2. **Limitations: “In addition, we decided to focus on the provision aspect of the quality of care framework, therefore, further research is needed to unravel the experience of home-based PNC measures.” if this was a choice of the authors then it falls beyond he scope of objectives and is not a limitation, but a recommendation for future research.**

We agree that this is more of a recommendation for future research than a limitation, as the authors intentionally chose to focus on the provision of care. Therefore, the following sentence was removed from the limitation section and added to the section on future perspectives (line 280, page 24): *“We decided to focus on the provision aspect of the quality of care framework, therefore, further research is needed to unravel the experience of home-based PNC measures.”*

3. **In the tables, the note defining the * is confusing because it refers to using the symbol when studies describe two different settings. It is difficult to distinguish which is which: “ Measures marked with * pertain to studies that include either exclusively home births or both facility-based births and home births”**

We understand the confusion, therefore, we have rephrased the sentence to *“Measures marked with * pertain to studies that also include home-births.”*

4. **The previous comment about breastfeeding support was to mean that in the table it currently falls under recommendation number 38 “responsive care to infants and children 0-3”, while there is recommendation n42 on breastfeeding which includes “Exclusive breastfeeding”.**

We differentiated the recommendation on exclusive breastfeeding from general breastfeeding support (such as promotion and education). Exclusive breastfeeding specifically means that no other food or liquids are given, while breastfeeding support encompasses a broader range of activities, including assistance with positioning and attachment. Therefore, we decided to categorize this recommendation under "responsive care."

5. **In the first paragraph of the discussion, authors introduce new findings related to frequency of PNC home visits – which I fail to see in the results while this is clearly a finding. I recommend to add this to results’ section. Additionally, the information on timing of PNC are listed in S3 but not mention in the results. Moreover, it is not clear how the study design could influence the number of PNC visits just from this sentence alone: “Within individual studies, the frequency of home visits varied according to the study design, ranging from one to 28 visits”. is this related to experimental studies? This should be clarified and a distinction should be made between the results from the guideline documents and those related to experimental studies.**

The findings related to frequency and timing of PNC home visits are now, in addition to the overview in S3, also included in the results of the review. On page 8, line 163 a small section was added under *‘Timing and frequency of home-based PNC visits’: “Both timing and frequency differed across different contexts. Within experimental studies, the frequency of home visits varied according to the study design, ranging from one to 28 visits. According to WHO recommendations, three to five PNC contacts are advised for facility-based deliveries, with an additional contact recommended for home births. With regard to timing, experimental studies display a wide range of intervals. A comprehensive overview of these timings is provided in S3 Table. The WHO recommendations have remained relatively consistent over the years. The most recent guidelines suggest scheduling a first visit between 48 and 72 hours postpartum. If the mother gave birth at home, a visit is recommended as early as possible within the first 24 hours postpartum. Finally, PNC should be offered until the 6th week postpartum according to the recommendations.”*

Moreover, the sentence “Within individual studies, the frequency of home visits varied according to the study design, ranging from one to 28 visits” is removed from the discussion section and added to the paragraph cited above (in the result section). "Individual studies” referred indeed to experimental studies. Consequently, we adapted the wording in this sentence to clarify the distinction between experimental studies and the guideline documents.

6. **PLOS authors have the option to publish the peer review history of their article (**[**what**](https://eur03.safelinks.protection.outlook.com/?url=https%3A%2F%2Fjournals.plos.org%2Fglobalpublichealth%2Fs%2Feditorial-and-peer-review-process%23loc-peer-review-history&data=05%7C02%7Cannsofie.mespreuve%40ugent.be%7C9c4740f6e7134d15270908dc9053c9f1%7Cd7811cdeecef496c8f91a1786241b99c%7C1%7C0%7C638543936761523531%7CUnknown%7CTWFpbGZsb3d8eyJWIjoiMC4wLjAwMDAiLCJQIjoiV2luMzIiLCJBTiI6Ik1haWwiLCJXVCI6Mn0%3D%7C0%7C%7C%7C&sdata=Jis2P8IvnlI6UuCAltXq8GDq%2F9YXRNjJRz3w7zF%2Fesc%3D&reserved=0) **does this mean?). If published, this will include your full peer review and any attached files.**
   We agree to publish the peer review history of this article.
